# Molecular Assessment of Genes Linked to Immune Response Traits of Honey Bees in Conventional and Organically Managed Apiaries

**DOI:** 10.3390/insects11090637

**Published:** 2020-09-17

**Authors:** Shalom C. Siebert, Lambert H. B. Kanga, Sheikh M. Basha, Jesusa C. Legaspi

**Affiliations:** 1 Center for Biological Control, College of Agriculture and Food Sciences, Florida A&M University, Tallahassee, FL 32307, USA; shalomsiebert@gmail.com (S.C.S.); mehboob.sheikh@famu.edu (S.M.B.); 2 Center for Medical, Agricultural and Veterinary Entomology, USDA-ARS, Tallahassee, FL 32308, USA; jesusa.legaspi@ars.usda.gov

**Keywords:** honey bee, genomic assessment, conventional and organic apiaries, immune response, Varroa mite

## Abstract

**Simple Summary:**

Honey bees play a critical role in agriculture as they provide pollination services to many agricultural crops. However, honey bee populations continue to decline due to exposure to pesticides, habitat destruction, pests, diseases and beekeeping practices. In this study, we assessed selected biological parameters associated with honey bee health in two beekeeping practices (organic and conventional). We compared total protein content in young worker bees from organically and conventionally managed apiaries. We also assessed differential gene expression at two levels of Varroa mite infestations (0% and 5%) in selected genes involved in nutrition and cellular defense (vitellogenin (Vg), malvolio (Mvl), prophenoloxidase (PPO)-, genes involved in lifespan (superoxide dismutase (Sod 1), superoxide dismutase 2 (Sod2)) and immune function genes (immune deficiency (Imd), spaetzle (Spz). Total soluble protein in young adult worker bees was similar in both beekeeping practices. The genes PPO, Vg and Mvl were upregulated in young adult bees with no mite infestations from organically managed apiaries and could mount an immune response through Spz and Sod 1 when challenged by 5% Varroa mite infestation levels. Overall, these findings provide useful insights into the genetic response of honey bees under two beekeeping practices and could help improve honey bee health.

**Abstract:**

Honey bees are of great economic importance, not only for honey production but also for crop pollination. However, honey bee populations continue to decline mainly due to exposure to pesticides, pathogens and beekeeping practices. In this study, total soluble protein was measured, total RNA was extracted and first-strand cDNAs were generated. Quantitative PCR was used to assess the relative expression (transcript abundances) of immune function-related genes in honey bees collected from organically and conventionally managed hives. Honey bees collected from conventionally managed hives with 0% Varroa mite infestation levels displayed an upregulated expression of the prophenoloxidase gene (cellular defense). Similarly, honey bees collected from organically managed hives had increased levels of the vitellogenin gene (immune function and longevity). The gene expression for malvolio (sucrose responsiveness) was highest in organically managed hives with 0% Varroa mite infestations. Young adult bees collected from organically managed hives with 5% Varroa mite infestation levels had upregulated expressions of the gene spaetzle, whereas bees from similarly infested, conventionally managed hives did not, suggesting that honey bees from organically managed hives could mount an immune response. In young adult bees collected from organically managed hives only, the expression of the immune deficiency gene (antimicrobial defense) was upregulated. The relative gene expression for superoxide dismutase 1 increased in young adult bees collected from hives with 5% Varroa mite infestation levels as expected. However, for superoxide dismutase 2, there was a high level of gene expression in adult bees from both conventionally managed hives with 0% Varroa mite infestation levels and organically managed hives with 5% Varroa mite infestations. The gene CYP9Q3 (pesticide detoxification) that metabolizes coumaphos and fluvalinate was upregulated in adult bees collected from organically managed bees. Overall, these findings provide useful insights into the genetic response of honey bees to some environmental stressors and could be an important component of best beekeeping practices that intend to enhance honey bee health.

## 1. Introduction

The availability of environmental nutrients is an important determining factor of an organism’s growth and survival; malnutrition can impair immune function and consequently affect animal health, resistance to diseases and survival [1]. Pollen and nectar are essential dietary sources for honey bees [2]. Pollen contains proteins, amino acids, lipids, vitamins and minerals, all of which are vital for individual honey bee health and colony growth and development [1,2]. Nectar, in the form of honey, is the carbohydrate source that provides the honey bee with energy [3]. Honey bees forage on a diversity of plants in order to receive adequate nutrition [1]. However, with the frequent placement of hives on monocultures, honey bees are faced with nutritional deficiencies affecting physiological development, immunocompetence (the ability to mount an immune response), or increased pesticide sensitivity [1,4]. The use of honey bees for pollination services has also increased honey bee exposure to harmful pesticides [5]. A combination of factors, including parasites, pathogens, pesticides and beekeeping practices, are the main factors causing the increased mortality of honey bee colonies [6]. The parasitic *Varroa destructor* (Anderson and Trueman, Varroa mite) is the major factor underlying colony loss in the U.S. and other countries [7,8]. Infestation by the Varroa mite contributes to stress and weakens honey bee colonies by directly affecting some genes encoding antimicrobial and immune responses (immune deficiency) [9]. High infestations of Varroa mites ultimately lead to the death of colonies lacking innate mite suppression behaviors [10] and Italian honey bees are known to be susceptible to infestations by Varroa mites [11]. Beekeeping practices (poor nutrition, in-hive miticide treatments and migratory practices) have devastating impacts on honey bee health and productivity [12]. In this study, we compared the activities of selected genes linked to honey bee health between conventionally and organically managed apiaries. In an organically managed apiary, beekeepers register with an appropriate certification agency (USDA Organic), comply with agency guidelines and set up a certifiable audit trail. These include no chemicals used to control pests and diseases and the availability of organically certified agricultural land for bee forage, beekeepers rely mainly on best management practices [13]. In conventionally managed apiaries, beekeepers use chemicals to control honey bee pests and diseases and also nonorganic feed such as corn syrup and sugar syrup. These chemicals include antibiotics for disease control, pyrethroids and organophosphate insecticides for pest control; synthetic wax foundations are also used. This study was intended to provide useful insights that may correlate with beekeeping practices.

## 2. Materials and Methods

### 2.1. Honey Bee Colonies and Honey Bee Samples

The honey bee samples were collected in four apiaries (two organically managed and two conventionally managed apiaries); each apiary contained more than 30 established honey bee colonies housed in Langstroth hive bodies. These pairs of apiaries (organic and conventional) were located in North Florida (Monticello, Wewahitchka) and south Florida (Loxahatchee). In each apiary, we established two subsets of five colonies with 0% and 5% levels of Varroa mite infestations. These levels of mite infestations were chosen as reference points because, in previous studies, we found that nearly all honey bee colonies with 5–10% Varroa mite infestations at the start of field trials perished before the end of the 42-day experimental period [14]. Thus, these two levels of mite infestations were used as benchmarks for comparison in this study. To establish the two levels of mite infestations, alcohol wash counts as described by Delaplane et al. [15] were conducted in the evening on 30 honey bee colonies in each conventionally and organically managed apiaries. The alcohol wash procedure involves brushing ≈300 bees from the edge of the brood chamber in each colony into a 1 L glass jar containing 70% ethanol. The jars were taken to the laboratory and shaken for 3–5 min to dislodge the mites from the bees and the alcohol solution was poured through a mesh screen into a white plastic tray to separate the bees from the mites. The procedure was repeated twice, or until there were no mites in solution. The bees were examined individually to ensure all mites were removed and counted. The number of mites in the sample were divided by the number of bees in the sample and recorded as mites per adult bee (a measure of Varroa mite infestations of adult bees). The five colonies in each level of Varroa mite infestations were placed into two circles separated by 25 m in the apiary, each colony was about 3 m from the next colony in the circle to reduce drifting of honey bees [16]. Samples of six newly emerging adult (nurse) worker bees per colony were obtained from the two Varroa mite infestation levels described above in organically and conventionally managed apiaries. To collect the bee samples from the colony with 0% Varroa infestations, the brood chamber was open, a frame with open brood was chosen, the frame was shaken off and six emerging young adult bees (nurse bees) on the brood without any Varroa mites were collected. We repeated the same procedure to collect samples from all five bee colonies in the group (level of Varroa mite infestations). To collect the bee samples from the 5% mite infestations, we used the same procedure, but six nurse bees carrying Varroa mites were collected from each of the five colonies in the group. Thus, a total of 30 young adult worker bees (n = 30) were collected early in the morning in each of the two levels of mite infestations in each apiary. These nurse bees have been fed by pollen and nectar and exposed to hive matrices during their 21-day development time inside the sealed brood. We also collected pollen, wax, honey and brood for residue analysis [17]. The bee samples were labeled per colony, management type, location and level of Varroa mite infestations. They were then placed in plastic crush-proof containers, held on dry ice and stored into a −80 °C freezer until used for the different experiments.

### 2.2. Protein Determination

Although several factors (diseases, parasites, pesticide exposure, nutrition and beekeeping practices) are involved in the decline of honey bee colonies, habitat, forage quality and nutritional stress have emerged as critical factors impacting honey bees and colony strength [18]. Smart et al. [19] reported that among the physiological markers evaluated, protein levels were most closely indicative of population size and colony robustness. Thus, in this experiment, we assessed total soluble protein in worker bees from the two beekeeping management types. Bicinchoninic acid protein (BCA) assays were used to quantify the total soluble protein content of adult worker bees from honey bee colonies in conventionally and organically managed apiaries. For this experiment, we had nine replicates of two young adult bees in the conventionally and 11 replicates in the organically managed apiaries. Two young adult worker bees from conventionally and organically managed apiaries were removed from the −80 °C freezer and placed on ice. One mL of TES extraction buffer (10 mM Tris-HCl, pH 7.6, 1 mM EDTA and 0.25 M sucrose) was added to the bees and homogenized with a mortar and pestle on ice. Samples were placed on ice for 30 min after which they were centrifuged at 14,000× *g* for 5 min. The supernatant was then transferred from each tube to a separate 0.5 microcentrifuge tube and frozen at −80 °C until further analysis. The BCA protein assay was performed by adding 95 µL of TES buffer, 5 µL thawed protein extract and 200 µL BCA working reagent to individual polymerase chain reaction (PCR) reaction tubes. The tubes were vortexed, spun and incubated for 30 min at 37 °C on a thermocycler. Tubes were then cooled on ice for 15 min and absorbance levels were recorded using a nanodrop spectrophotometer. Concentrations of protein in tissue homogenates were estimated by the methods of Bradford [20] with bovine serum albumin as the standard.

### 2.3. RNA Extraction and CDNA Synthesis

Eight individual nurse bees from each of the two levels of mite infestations (0% and 5%) in organically and conventionally managed apiaries were used in experiment runs. A sample of 16 young adult bees was used at each level of Varroa mite infestations under each beekeeping management regime. Each bee was individually grounded in liquid nitrogen and total RNA was extracted using the RNeasy Mini Kit (Qiagen; www.qiagen.com) according to the manufacturer’s protocol. The total RNA solution was treated in liquid RNase-Free DNase I and purified on column RNA Cleanup and DNase digestion (Qiagen) according to the manufacturer’s protocol. Total RNA was quantified using a nanodrop spectrophotometer. RNA quality was checked by agarose gel electrophoresis. First-strand cDNA was synthesized from 1 µg total RNA using a master mix containing 4 µL of Transcriptor RT reaction buffer, 0.5 µL of Protector RNase inhibitor, 2 µL of DNA mix, 0.5 µL of transcriptor reverse transcriptase and 1 µL of anchored-oligo (dT)18 Primer. Synthesis was carried out at 55 °C for 30 min followed by 5 min at 85 °C.

### 2.4. Primer Design

To assess the potential impact on the primary function of genes involved in honey bee health, we selected a subset of known genes from the literature [1,21,22,23,24]. These include genes involved in nutrition and cellular defense [vitellogenin (Vg), malvolio (Mvl), prophenoloxidase (PPO)], two genes involved in lifespan (superoxide dismutase (Sod 1), superoxide dismutase 2 (Sod2) and two immune function genes (immune deficiency (Imd), spaetzle (Spz)). The CYP9Q3 gene was also selected because it is induced by p-coumaric in hive matrices (honey, pollen and propolis) and is used to metabolize chemicals found in honey and pollen, as well as acaricides (coumaphos and fluvalinate), used in-hive for the management of Varroa mite [24] (Table 1). Primers were designed using information from the National Center for Biotechnology Information, (http://www.ncbi.nlm.nih.gov, NCBI) and Bee Base (http://hymenopteragenome.org). Primer conditions were optimized by determining the optimal annealing temperature (Ta) and primer concentration [25]. Before real-time PCR (RT-PCR) each primer was validated by specific PCR amplifications and 1.5% agarose gel electrophoresis in TBE buffer and ethidium bromide staining. 

### 2.5. Real-Time Quantitative Polymerase Chain Reaction

Real-time quantitative PCR amplification was performed in individual nurse bees collected as described above from the two beekeeping practices in a 20 µL reaction mixture using SYBR Green Supermix (Bio-Rad Laboratories). The oligonucleotide amplification primers are listed in Table 1. The PCR reactions were carried out in 96-well microliter plates using a Bio-Rad thermal cycler (Bio-Rad Corp.). The amplification was programmed as follows: initial 2 min UDG (uracil-DNA glycosylases) incubation step at 50 °C, 2 min denaturation at 95 °C, followed by 45 cycles of denaturation at 95 °C for 20 s and 40 s annealing at the optimal annealing temperature (Ta) at which fluorescence was measured [25]. The amplification results were expressed as a threshold cycle (CT) value. Relative quantifications were calculated by using threshold cycle numbers for the target gene by subtracting from the reference gene (β-actin) for each sample. Relative expression (RQ) of the gene was calculated by raising 2 to the negative power of the difference in CT values [26].

### 2.6. Data Analysis

The amount of total soluble protein and total RNA per bee in conventionally and organically managed colonies was analyzed using a general linear model (PROC GLM) [27]. Means between treatment groups were separated using Tukey’s studentized range test [27]. Data on the relative expression of genes (transcript abundances) for bee samples at the different levels of Varroa mite infestations in conventionally and organically managed colonies were subjected to (PROC MIX) [27]. Treatments were modeled as fixed effects; type of management and type of management–by-treatment interactions were modeled as random effects. When necessary the data on relative expression were transformed to log (x + 1) to satisfy the assumptions of normality before analysis. When the mean values were similar between samples collected from North and South Florida under each management regime (organic and conventional), the data were pooled to provide a better estimate of the statistics. A significance level of alpha = 0.05 was used for all statistical tests.

## 3. Results

### 3.1. Honey Bee Protein and RNA Contents

**Figure 1:**
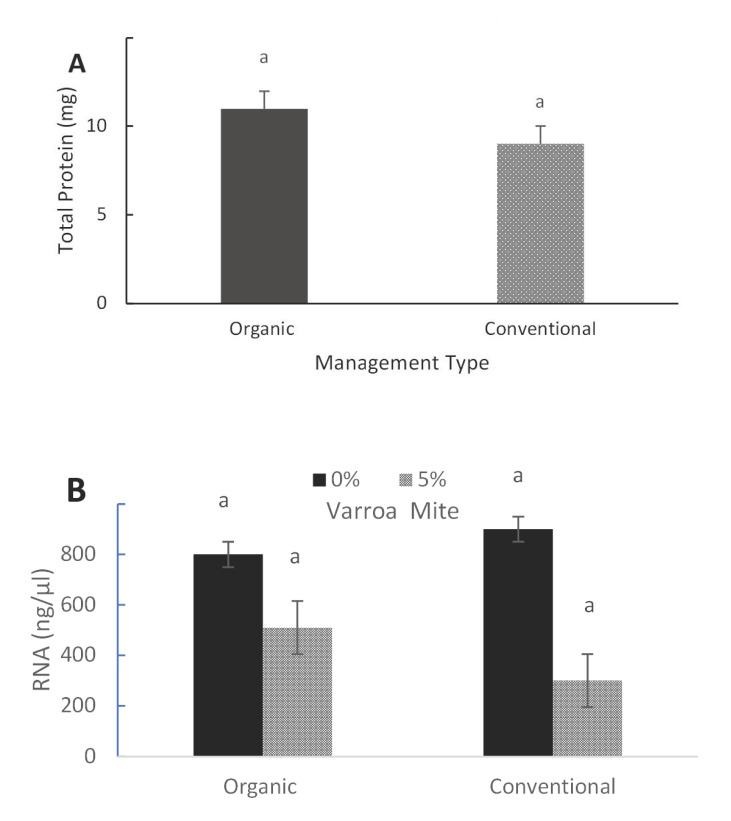
Protein in young adult bees from organically and conventionally managed apiaries. Data on the total soluble protein content of adult bees between the two beekeeping practices were pooled. (**A**) Mean (±SE) total soluble protein and (**B**) mean total RNA compared by Varroa mite infestation levels. Different letters above bars indicate treatment differences (*p* < 0.05, Tukey’s test).

The total soluble protein content of young adult bees from conventionally managed apiaries was similar to that from organically managed apiaries (*F* = 1.36; *d.f.* = 1, 17; *p* = 0.263; Figure 1A). Similarly, total RNA absorbance values did not differ between bees collected from conventionally or organically managed apiaries with 0% Varroa mite infestations (*F* = 3.225; *d.f.* = 3, 3; *p* = 0.826). However, the total RNA absorbance values from bees collected from conventionally managed hives with 5% Varroa mite infestations were relatively lower compared to those of organically managed hives (*F* = 3.098; *d.f.* = 3, 3; *p* = 0.250; Figure 1B).

### 3.2. Gene Expression

**Figure 2:**
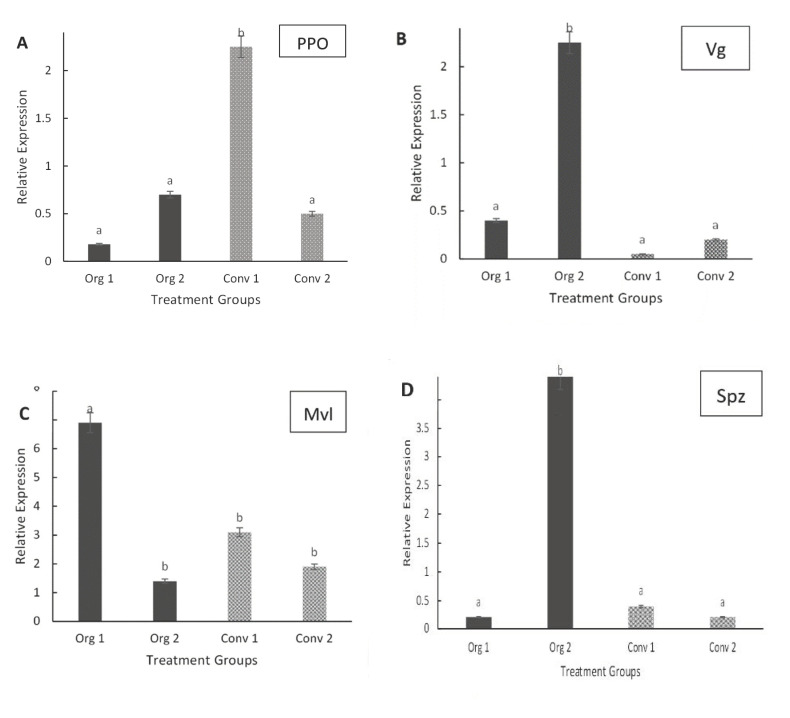
Mean (±SE) relative gene expression in young adult bees collected from: organically managed hives with Varroa mite infestation levels of 0% (Org 1) or 5% (Org 2) and conventionally managed hives with Varroa mite infestation levels of 0% (Conv 1) or 5% (Conv 2). Relative expression data were normalized to β-actin. Different letters above bars indicate treatment differences (*p* < 0.05, Tukey’s test). (**A**) Prophenoloxidase. (**B**) Vitellogenin. (**C**) Malvolio. (**D**) Spaetzle.

**Figure 3:**
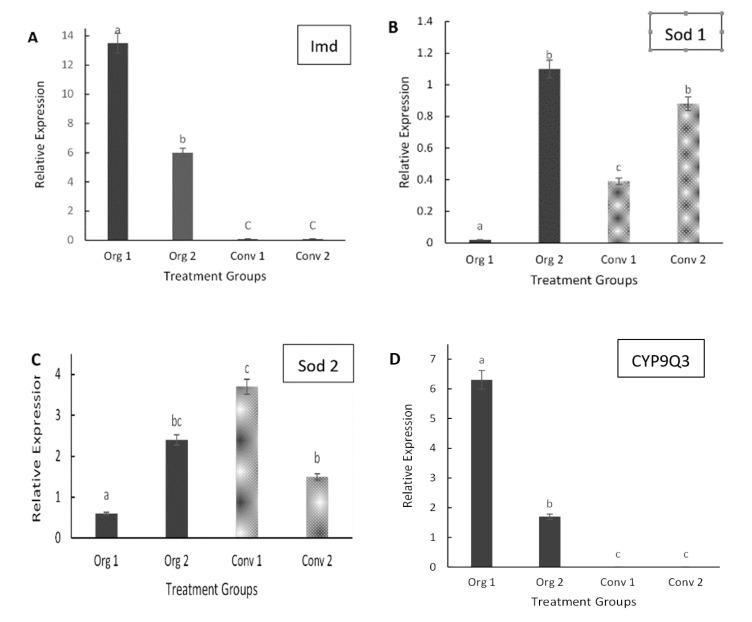
Mean (±SE) relative gene expression in young adult bees collected from: organically managed hives with Varroa mite infestation levels of 0% (Org 1) or 5% (Org 2) and conventionally managed hives with Varroa mite infestation levels of 0% (Conv 1) or 5% (Conv 2). Relative expression data were normalized to β-actin. Different letters above bars indicate treatment differences (*p* < 0.05, Tukey’s test). (**A**) Immune deficiency gene. (**B**) Superoxide dismutase 1. (**C**) Superoxide dismutase 2. (**D**) CYP9Q3.

The relative gene expression levels (transcript abundances) of PPO in young adult bees from conventionally managed hives with 0% Varroa mite infestations were significantly upregulated (*F* = 78.43; *d.f*. = 1, 14; *p* < 0.0001) compared to bees from organically managed hives, but there were no significant differences in PPO gene expression in bees with 5% Varroa mite infestations under either management practice (Figure 2A). The effect of management type (*F* = 5.56; *d.f.* = 1, 14; *p* = 0.0157) and the management type-by treatment interactions (*F* = 33.51, *d.f*.= 1, 14; *p* < 0.0001) were statistically significant in regard to the PPO gene expression levels. Vitellogenin(Vg) gene expression levels were significantly higher (*p* < 0.0001) in bees from mite-infested (5% infestation levels) organically managed hives compared to bees from conventional hives with 0% Varroa mite infestations or to bees from either level of mite infestation in conventionally managed hives. Transcript abundances for Vg in bees collected from conventionally managed hives were generally low (Figure 2B). The gene expression level of malvolio (Mvl) in honey bees from organically managed hives (with 0% Varroa mite infestations) was significantly higher (*p* < 0.0001) than that from mite-infested organically managed hives or transcript levels for Mvl were similar between the two densities of Varroa mite infestations in conventionally managed hives (Figure 2C). The expression levels of the gene spaetzle (Spz) was significantly increased only in bees from organically managed hives with 5% Varroa mite infestations, but not in hives with 0% mite infestations hives nor in conventionally managed hives with or without Varroa mites. Transcript levels for Spz were similar between the two densities of Varroa mite infestations in conventionally managed hives (Figure 2D). Bees from all organically managed hives displayed significantly higher expression levels of the immune deficiency gene (Imd) when compared to conventionally managed hives. Transcript levels for Imd were very low in conventionally managed hives (Figure 3A). In hives with 0% Varroa mite infestations, expression levels of the gene superoxide dismutase 1 (Sod 1) were lower in adult bees from organically managed hives than those from conventionally managed hives (Figure 3B). Transcript levels for Sod 1 were upregulated but similar in both management regimes at 5% Varroa mite infestations. For the gene superoxide dismutase 2 (Sod 2), there were significant differences (*p* < 0.0001) in transcript abundances between adult bees collected from organically managed and conventionally managed hives at 0% Varroa infestation levels. However, transcript levels of Sod 2 were similar in bees collected from organically managed hives (with 5% Varroa infestations) compared to those from conventionally managed hives (Figure 3C). Bees from organically managed hives displayed significantly higher transcript levels of CYP9Q3 than those from conventionally managed hives at both 0% and 5% Varroa mite infestations. The differences were higher in hives with 0% Varroa mite infestation levels (Figure 3D).

## 4. Discussion

In this study, we showed that some selected genes linked to honey bee health can be affected by beekeeping practices. There were no significant differences in total soluble protein and total RNA between adult bees collected from organically and conventionally managed hives. Data from quantitative PCR for the detection of specific gene expressions indicated an elevated defense level of PPO in honey bees from conventionally managed hives at 0% Varroa mite infestations of adult bees. These findings suggested that these honey bees may have been stressed due to unknown factors (which may have included exposure to pesticides, pathogens and food sources) [28]; however, there was little difference in transcript levels of PPO between hives infested with Varroa mites whether managed organically or conventionally. Gregorc et al. [29] found that the prophenoloxidase-activating enzyme is induced with diet-acquired pesticides, consistent with an earlier study where we found that the pesticide loads were highest in pollen and wax from conventionally managed apiaries [17]. Thus, our data suggested that PPO transcript induction may result from the combinations of diet-acquired pesticides and other environmental stressors.

Under both management practices, gene expression levels of Vg increased 4.5-fold when bees were under parasitic attack (5% Varroa mite infestations) in organically managed hives, a trend somewhat unexpected; however, this situation may be due to the exposure of young adult bees to pesticide residues in wax foundations and in pollen diets [17,30]. Vitellogenenin is also involved in many physiological processes that include immune senescence and longevity [22]. The organophosphate coumaphos and the botanical derivative thymol (Apiguard) have been found to alter metabolic responses including the downregulation of Vg [22], thus, our data may suggest a shorter lifespan of adult bees from conventionally managed hives in this study.

Young adult bees from organically managed hives with 0% Varroa mite infestations had the highest Mvl gene expression, which suggested that these bees have a higher foraging capacity and sucrose responsiveness of these bees which may be due to lower exposure to pesticides. Ben-Sharer et al. [31] reported that workers foraging on pollen have a high level of Mvl in the brain. The Varroa mite is known to suppress immune function [9,29]; thus, the low levels of Mvl transcripts in young adult bees from 5% Varroa mite infestations under either organically or conventionally managed hives may indicate a negative impact of the parasitic Varroa mite on the foraging behavior of these bees. In addition, some pesticides such as the pyrethroid fluvalinate and the neonicotinoid imidacloprid have been found to reduce sucrose responsiveness by decreasing the sensitivity of honey bees to sucrose concentrations [23,32].

The gene Spz is a member of the immune signaling pathway against pathogens and is upregulated in infected larvae in order to mount an immune response [1,33]. Evans et al. [33] reported that Spz transcripts increased in honey bees fed polyfloral pollen diets but decreased in the presence of Varroa mite. This is not consistent with our results, but the increase in Spz transcripts might suggest that parasitized bees from organically managed hives (5% Varroa infestations) were better able to mount an immune response when they were challenged by Varroa mites. However, transcript levels of Spz decreased in young adult bees collected from conventionally managed hives at higher (5%) Varroa mite infestations.

Our data indicated an upregulated Imd gene in bees from organically managed hives but the absence of transcripts of Imd in young adult bees collected from conventionally managed hives; whether or not these findings are linked to beekeeping practices remain to be investigated.

Insects suffer oxidative stress under environmental factors from pathogens; however, antioxidant enzymes such as Sod 1 provide some defense [21]. Therefore, the upregulation in Sod 1 and 2 in honey bees from high Varroa-mite-infested hives was expected. The upregulation of Sod (especially Sod 2) in honey bees from conventionally managed hives without mite infestations could suggest the presence of additional environmental stressors (pesticides, food quality, diseases and beekeeping practices).

There were notable differences in transcript levels of CYP9Q3 between bees from organically managed hives compared to conventionally managed hives. This gene is important in the detoxification of pesticides, especially coumaphos and fluvalinate [24]. Mao et al. [24] found that *p*-coumaric acid, a constituent of pollen, had a major role in the upregulation of CYP9Q3. This may explain why the organically managed honey bees possessed high expression levels of this detoxification gene. Our data suggested that the combination of exposure to pesticides and high levels of Varroa mite infestations has substantial detrimental effects on the expression of CYP9Q3 in conventionally managed hives.

Our overall data provided useful insights on the potential effects at the gene transcriptional levels of honey bees under organically or conventionally managed apiaries. The statistical significance of the effect of management type and management type–by-treatment interactions suggested that differential gene expression was affected by beekeeping practices. Exposure of honey bees to pesticides in the field and in-hive treatments along with the Varroa mite infestations significantly affected the expression of genes related to honey bee health. The combined effects of pesticide and Varroa mite pressures were not assessed in this experiment, but it could be of great interest in future studies by providing useful information on the interactions between these stressors on honey bee health.

## 5. Conclusions

Bee health is critical for the success of pollination-based agriculture, which produces about a third of the diet in the United States. A healthy and secure honey bee industry is valuable to modern agriculture, providing pollination services for more than 90 commercial crops. Unfortunately, the number of honey bee colonies in the U.S. has been declining over the years. A combination of causal factors, including parasites, pathogens, pesticides and beekeeping practices, are cited as responsible for the increased colony mortality. In this study, we assessed the immune response to the impact associated with the most important parasite (Varroa mite) of honey bee colonies in two beekeeping practices. Our data suggested the levels of Varroa mite infestations negatively affected the expression of genes linked to honey bee health. The overall results provide useful insights into the development of strategies that promote honey bee health and sustainable beekeeping.

## Figures and Tables

**Table 1:** Primers used for quantitative PCR of honey bee immune-link genes.

Gene	BeeBase *	Forward Primer (5′ to 3′)	Reverse Primer (5’ to 3’)	Ta (°C)	Function
Prophenoloxidase (PPO)	GB18313- RA	GGA CAT CAA TCG ACA AGT TG	GAC GTC GAT TCC ATT TTT CT	52	Catalyzes cellular defense [24]
Vitellogenin (Vg)	GB13999	CAC ATC CCT CGC CCT TCC AA	CAC GTA TCC CTC GTT GTC CAT	51	Immune function and longevity [24]
Malvolio (Mvl)		CCT TGG TAT AAA GAT TAT GAC AGG AAT ATG	CAA GAG CAC TGT GAA GAT ACA AGT TAT G	57	Involved in sucrose responsiveness [1,22]
Spaetzle (Spz)	GB 15688	TGC ACA AAT TGT TTT TCC TGA	GTC GTC CAT GAA ATC GAT CC	49	Member of the Toll-immune signaling pathway against fungi and bacteria [1,23]
Superoxide dismutase 1 (Sod)	GB 10133	GAC TAA AGC AGT GTG CGT TC	TTA TCG CCA AAT TCA TGA AC	52	Codes for antioxidants; increase lifespan [1]
Superoxide mutase 2 (Sod 2)	GB14346	CAA TGT TTG CAG CAA GAC GT	GTT GCA TGG TGC TTT GAA TG	53	Codes for antioxidants; increases lifespan [1]
Immune deficiency (Imd)	GB 18606	TGT TAA CGA CCG ATG CAA AA	CAT CGC TCT TTT CGG ATG TT	50	Antimicrobial defense [1,22]
CYP9Q3	GB19967	GTTCCGGGAAAATGACTAC	GGTCAAAATGGTGGTGAC	53	Pesticide detoxification [21]

* BeeBase accession number.
